# An *In Situ* Autologous Tumor Vaccination with Combined Radiation Therapy and TLR9 Agonist Therapy

**DOI:** 10.1371/journal.pone.0038111

**Published:** 2012-05-30

**Authors:** Huagang Zhang, Laibin Liu, Dong Yu, Ekambar R. Kandimalla, Hui Bin Sun, Sudhir Agrawal, Chandan Guha

**Affiliations:** 1 Department of Radiation Oncology, Albert Einstein College of Medicine and Montefiore Medical Center, Bronx, New York, United States of America; 2 Department of Orthopedics, Albert Einstein College of Medicine and Montefiore Medical Center, Bronx, New York, United States of America; 3 Department of Pathology, Albert Einstein College of Medicine and Montefiore Medical Center, Bronx, New York, United States of America; 4 Idera Pharmaceuticals, Inc., Cambridge, Massachusetts, United States of America; Cedars-Sinai Medical Center, United States of America

## Abstract

**Purpose:**

Recent studies have shown that a new generation of synthetic agonist of Toll-like receptor (TLR) 9 consisting a 3′-3′-attached structure and a dCp7-deaza-dG dinucultodie shows more potent immunostimulatory effects in both mouse and human than conventional CpG oligonucleotides. Radiation therapy (RT) provides a source of tumor antigens that are released from dying, irradiated, tumor cells without causing systemic immunosuppression. We, therefore, examined effect of combining RT with a designer synthetic agonist of TLR9 on anti-tumoral immunity, primary tumor growth retardation and metastases in a murine model of lung cancer.

**Methods:**

Grouped C57BL/6 and congenic B cell deficient mice (B^−/−^) bearing footpad 3LL tumors were treated with PBS, TLR9 agonist, control oligonucelotide, RT or the combination of RT and TLR9 agonist. Immune phenotype of splenocytes and serum IFN-γ and IL-10 levels were analyzed by FACS and ELISA, 24 h after treatment. Tumor growth, lung metastases and survival rate were monitored and tumor specific antibodies in serum and deposition in tumor tissue were measured by ELISA and immunofluorescence.

**Results:**

TLR9 agonist expanded and activated B cells and plasmacytoid dendritic cells in wild-type mice and natural killer DCs (NKDCs) in B cell-deficient (B***^−/−^***) mice bearing ectopic Lewis lung adenocarcinoma (3LL). Combined RT with TLR9 agonist treatment inhibited 3LL tumor growth in both wild type and B***^−/−^*** mice. A strong tumor-specific humoral immune response (titer: 1/3200) with deposition of mouse IgG auto-antibodies in tumor tissue were found in wildtype mice, whereas the number of tumor infiltrating NKDCs increased in B^−/−^ mice following RT+ TLR9 agonist therapy. Furthermore, mice receiving combination therapy had fewer lung metastases and a higher survival than single treatment cohorts.

**Conclusions:**

Combination therapy with TLR9 agonist and RT induces systemic anti-tumoral humoral response, augments tumoral infiltration of NKDCs, reduces pulmonary metastases and improves survival in a murine model of 3LL cancer.

## Introduction

Ionizing radiation therapy (RT) has been used as a standard treatment modality for many solid tumors [1]. While tumoricidal properties of RT are instrumental for standard clinical application of RT, recent preclinical [2]–[4] and clinical studies [5] have applied immunomodulatory effects of RT. RT has been shown to increase the immunogenicity of tumor cells by amplifying the tumor-specific peptide repertoire [6] and upregulating cell surface expression of MHC determinants and costimulatory molecules [7]. Furthermore, RT modifies the tumor microenvironment by enhancing the release of CXCL16 from tumor cells [8] and upregulating VCAM-1 on the tumor vasculature [9] to favor the recruitment and trafficking of tumor specific cytotoxic T cells to tumor tissue. RT also induces the expression of cell surface, death receptor, Fas, thereby, increasing the susceptibility of irradiated tumor cells to T cell-mediated killing [10]. These important findings indicate that RT could be combined with immunotherapy to improve the control of both localized and systemic tumor progression [11].

Toll-like receptor (TLR) agonists have been widely used in cancer therapy due to its ability of inducing potent anti-tumor immune response [12]. The structure of these agonists contains highly conserved molecular patterns common to cell surface and nuclear molecules in pathogens, termed pathogen-associated molecular patterns (PAMP) [13]. Binding of these ligands to TLRs triggers the activation of intracellular signaling pathways through nuclear factor kB (NF-kB) and mitogen-activated protein kinases and results not only in the activation of innate effector cells but also in the induction of adaptive immune response [14]. Among the fifteen TLR family members, TLR9 receptors recognize unmethylated cytosine-phosphate-guanosine (CpG) dinucleotides from bacterial DNA. TLR9 have been studied most extensively due to its ability of inducing adaptive cellular and humoral immune response. In mouse and human, TLR9 is only expressed on plasmacytoid dendritic cells (pDCs), B cells [15] and natural killer dendritic cells (NKDCs) [16], [17]. TLR9 agonists activate pDCs to secrete type I interferon (IFN) and induce maturation by upregulating the expression of co-stimulatory molecules such as CD80 and CD86 [18]. This initiates a range of secondary effects with eventual activation of natural killer (NK) cells and expansion of cytotoxic T lymphocytes (CTLs). TLR9 agonists also enhance the differentiation of B cells into antibody-secreting plasma cells and could potentially eradicate tumor cells through antibody dependent cellular cytotoxicity (ADCC) [19]–[21]. Furthermore, recent study has shown that TLR9 agonist dramatically increases the number of NKDCs in vivo and induces NKDCs to secrete IFN-γ via the autocrine effects of IL-12 [16]. The unique ability of NKDCs to directly lyse tumor cells provides another anti-tumor pathway mediated by TLR9 agonist.

Most of the oligodeoxynucleotide (ODN) agonists for TLR9 contain unmethylated CpG dinucleotides motifs, which are relatively common in bacterial DNA. CpG DNA has shown efficacy in the treatment of various cancers in mouse models but exhibited poor efficacy in primate system, including human clinical trial [22]. This has led to the discovery of new ODN by synthesizing a cytosine-phosphate-2′-deoxy- 7-deazaguanosine dinucleotide (CpR) motifs which showed more potent stimulating effect in mouse, monkeys and human [23]. This new generation of TLR9 agonist induces different cytokine profiles (higher or similar levels of IL-12 and lower IL-6) as compared with CpG ODN [24]. Recent studies have shown that these novel agonists of TLR9 induced mucosal Th1 immune responses in mouse [25], increased the sensitivity of human non small cell lung cancer (NSCLC) cell lines to chemotherapy [26] and inhibited the growth of murine CT26 colon tumor, B16.F0 melanoma and human breast cancers in vivo [27].

Although many preclinical studies utilizing TLR9 agonists have induced strong anti-tumoral immune response, TLR9 agonist is unable to control macroscopic primary tumors. Thus TLR9 agonists are considered as an adjuvant treatment for cancer patients that can be combined with traditional local therapies, such as, RT. Radiation therapy has great advantages over other traditional therapies such as chemotherapy because of the absence of systemic immunosuppression while presenting a source of tumor antigens, released from irradiated, dying tumor cells. This focal effect of RT enables the potential of a strong synergistic therapeutic effect if combined with immunostimulatory agents, such as TLR9 agonists. TLR9 agonists, CpG ODNs, have been combined with RT in various preclinical tumor models, such as, lung, melanoma and fibrosarcoma [28]–[31]. However, most experiments were performed with standard CpG oligonucleotides that have been shown to be highly active in murine models, while showing limited activity in humans. Previous reports also failed to characterize the cellular elements that mediate the anti-tumoral immunity after combination therapies with TLR9 and RT. We, therefore, examined the effect of combining RT with a designer TLR9 agonist that have been specifically developed to bind and activate primate and mouse TLR9 receptors. In this study, we have shown that combined RT and TLR9 agonist administration has synergistic anti-tumor effect in mice bearing a murine lung adenocarcinoma with improved primary tumor growth retardation and reduction of systemic pulmonary metastases. We demonstrate that two pathways could mediate this effect. First, RT enhances the generation of tumor-specific antibodies, possibly by amplifying the release of “tumor-specific” antigen pool for antigen presenting cells (APCs), such as, plasmacytoid dendritic cells (pDCs) and B-lymphocytes, while TLR9 agonists provide the activating signal for adaptive anti-tumoral immunity. Second, RT increases the penetration of antibodies and innate effector cells (NKDCs) into the tumor tissue by modifying tumor microenvironment.

## Materials and Methods

### Mice and Tumor Cell Line

Male C57BL/6J (National Cancer Institute, Bethesda, MD) and congenic B cell deficient mice (Igh-6^tm1Cgn^, The Jackson Laboratory, Bar Harbor, ME), 4–6 weeks of age, were bred and maintained at the Animal Resource Facility at Albert Einstein College of Medicine. All the animal protocols were approved by Institutional Animal Care committee and were performed following facility guidelines. Lewis lung carcinoma (3LL) cells (American Type Culture Collection, Manassas, VA) was propagated in high glucose DMEM supplemented with 10% FBS, sodium pyruvate, non-essential amino acids and 100 U/ml of penicillin and streptomycin (Invitrogen, Carlsbad, CA). Cells were maintained in a 37°C humidified incubator under 5% CO_2_.

### TLR9 Agonist

A CpR containing TLR9 agonist and a control oligonucleotide with CpC substitution, were synthesized at Idera Pharmaceuticals as described previously [24], [32]. The final product was 95% pure and endotoxin was less than 0.1 EU/mL by the Limulus assay (Bio-Whittaker **Lonza Walkersville Inc.,** Walkersville, MD). TLR9 agonist and the control oligonucleotide were dissolved in PBS at the concentration of 2 mg/ml and stored at −20°C until use.

### Experimental Design

**Figure 1:**
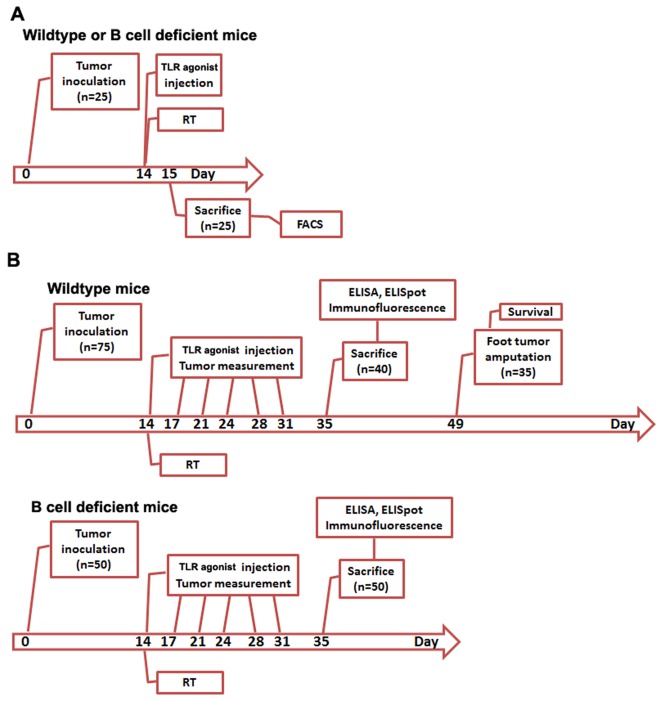
Experimental schema. The immunomodulatory effect of TLR agonist and RT was tested in both short term (A) and long term animal studies (B). Experiments were carried out on both C57BL/6 wildtype and cogenic B cell deficient mice. Mice were inoculated with 1×10^5^ 3LL cells on day 0 and were divided into 5 groups (n = 15/group for wildtype and n = 10/group for B cell deficient mice) that received either no treatment, 20 Gy RT of the primary tumor on day 14 post-tumor inoculation, only control oligo, only TLR9 agonist, or the combination of tumor RT, followed immediately by TLR9 agonist injection on day 14. For short term studies, 25 wildtype or B cell deficient mice were sacrificed on day 15 and splenocytes were harvested for flow cytometry. For long term studies, 75 wildtype and 50 B cell deficient mice were used. TLR9 agonist was injected subcutaneously, twice a week for three weeks (from day 14 to 35). The tumor volume was measured at 1–3-day intervals with a vernier caliper. Of the 75 wildtype mice, 40 mice were sacrificed on day 35 for immunological studies and the remaining 35 mice were used for survival studies. For B cell deficient mice, all of them were sacrificed on day 35 and were used for immunological studies.

The immunomodulatory effect of TLR agonist and RT was tested in both short term and long term animal studies. Mice were inoculated with 1×10^5^ 3LL cells on day 0 and were divided into 5 groups that received either no treatment, 20 Gy radiation of the tumor tissue, only control oligo, only TLR9 agonist, or the combination of irradiation of the tumor, followed by TLR9 agonist injection on day 14. For analysis of short term effect on splenoctyes, both wildtype and B cell deficient mice (n = 25 each) were sacrificed on day 15 and the phenotype of splenoctyes was analyzed by flow cytometry (Figure 1A). For analysis of long term effect on tumor growth and immune response, 40 wild type and 50 B cell deficient mice received different treatments were sacrificed on day 35 and splenocytes, tumor tissue and sera were collected for the use of immunological studies (ELISA, ELISpot and Immunofluorescence. Figure 1B). Thirty five wildtype mice were kept alive and were used for survival studies after tumor amputation on day 49. Detailed treatment procedure is explained in each method section.

### Phenotypic Analysis of Splenocytes by Flow Cytometry

Monoclonal antibodies specific for CD3, CD4, CD8, CD69, B220, NK1.1 and CD11c were obtained from BD bioscience (San Jose, CA). Phenotypic analysis of splenocytes in mice was performed 24 hours after IR and/or TLR9 agonist injection. Mice were anesthetized and then spleen was removed and minced in 2 ml of PBS and ground with a plunger. The cell suspension was passed through a 70 micron cell strainer (BD bioscience, San Jose, CA, USA) and erythrocytes were removed by hemolysis using a lysis buffer (BD bioscience, San Jose, CA, USA). Cells were then stained with the above monoclonal antibodies and analyzed by FACScan flow cytometer with Cellquest software. The relative proportions of lymphocyte subpopulations were determined as percentages of the total numbers of cells in a lymphogate defined by forward and side scatter properties.

### Detection of Serum Cytokine Levels by ELISA

Serum cytokine levels in mice treated with TLR9 agonist were detected by a commercial Th1/Th2 ELISA kit (eBioscience, San Diego, CA, USA). Briefly, serum samples were diluted 4 times with assay buffer and added into plates that were precoated with capture antibody. After 2 hours’ incubation, plates were washed 5 times with PBS-T and incubated with detection antibody followed by strepavidin-HRP. Plates were developed with TMB substrate for 30 minutes at room temperature, and the reaction was stopped by adding 1M H_2_SO_4_. Plates were read at 450 nm and concentrations were calculated according to the manufacturer’s instructions.

### Analysis of Antibodies and Lymphocytes Infiltration by Immunofluorescence

Two days after RT and/or TLR9 agonist injection, mice were sacrificed and tumors were surgically excised and embedded in tissue freezing medium (Electron Microscopy Sciences, Hatfield, PA, USA). Frozen tumor tissues were then sectioned at 5 mm thickness and stored in −20°C until use. For staining the NKDC infiltration in the tumor tissue, cryostat sections were aired dried, fixed in acetone and stained with anti-mouse CD11c monoclonal antibody (eBioscience, San Diego, CA, USA). After washing, sections were incubated with FITC-conjugated anti-hamster IgG (eBioscience, San Diego, CA, USA) and PE-CY7-conjugated anti-mouse NK1.1. For detecting the antibody infiltration, sections were stained with FITC-conjugated rabbit anti-mouse IgG. After 30 minutes incubation, sections were washed and covered by hardset mounting medium with DAPI (Vector Laboratories, Burlingame, CA, USA). Sections were subsequently visualized under a Leica SP2 AOBS Confocal microscope (Leica Microsystems, Bannockburn, IL, USA).

### Detection of Serum Tumor Specific Antibodies by Indirect ELISA

Titers of tumor specific antibodies in serum were measured by indirect ELISA assay as described previously [33]. Microtitre plates (eBioscience, San Diego, CA, USA) were coated with 10 µg/ml protein from 3LL cell lysate in coating buffer at 4°C overnight. The wells were blocked with 3% BSA in PBS (pH 7.2) at room temperature for 1 hour. Serum was incubated at two-fold serial dilution (from 1∶100 to 1∶12800) at room temperature for 3 hours. Plates were washed in PBS-Tween (0.05%) twice for 10 min each and incubated with goat anti-mouse IgG-HRP conjugate (1∶2000, BD bioscience, San Jose, CA, USA) at room temperature for 1 h. Plates were washed with PBS-T and developed with TMD substrate (eBioscience, San Diego, CA, USA) for 30 minutes at room temperature in the dark. The reaction was stopped with an acid stop solution and plates were read at 450 nm on ELISA reader.

The total IgG titer in mouse serum was measured by an Easy-Titer Mouse IgG Assay Kit (Thermo Fisher Scientific, Rockford, IL). Briefly, serum samples were incubated with anti IgG-sensitized beads for 5 minutes at room temperature and the reaction was stopped by adding equal volume of blocking buffer to the mixture. The absorbance was measured at 405 nm or 340 nm and sample total IgG titer was calculated according to the manufacturer’s instructions.

### Detection of Spleen Tumor Specific T Cells by ELISPOT Assay

The IFN-γ release ELISPOT assay was performed as described previously with slight modifications, using a commercial kit (MABTECH, Stockholm, Sweden). Plates (Millipore, Bedford, MA, USA) were coated overnight with anti-mouse IFN-γ monoclonal antibody (clone: AN18, 15 mg/ml) and washed six times. After blocking with 10% FBS, fresh isolated spleenocytes (2.5×10^5^ cells per well) were added together with RPMI 1640 medium, PMA (5 ng/ml) and Ionomycin (500 ng/ml), irradiated 3LL cells (20 Gy, 2×10^4^ cells per well), MUT1 peptide (FEQNTAQP, 10 mg/ml) respectively. After incubation for 40 hours at 37°C, cells were removed, and the plates were developed with a second (biotinylated) antibody (clone: R4-6A2, 1 mg/ml) to mouse IFN-γ and streptavidin-alkaline phosphatase. Plates were developed with BCIP/NBT substrate (Sigma-Aldrich, St. Louis, MO, USA) for 20 minutes at room temperature in the dark, and the reaction was stopped by rinsing plates with tap water. The membranes were air dried, and spots were counted using an automatic ELISOT reader (Autoimmun Diagnostika, Strassberg, Germany).

### Generation of Tumor Growth Curve, Enumeration of Lung Metastases and Survival Analysis

A total of 75 wildtype and 50 B cell deficient mice were injected subcutaneously with 1×10^5^ 3LL cells on the dorsum of right foot. Fourteen days after tumor transplant, mice were divided into 5 groups (n = 15/group for wildtype and n = 10/group for B cell deficient mice) that received either no treatment, 20 Gy radiation of the tumor tissue, only control oligo, only TLR9 agonist, or the combination of irradiation of the tumor, followed by TLR9 agonist injection. The irradiation was performed using a 40 MGC Philips orthovoltage unit operating at 320 kVp, 5 mA, and 0.5 mm copper filtration (2.60 Gy/min exposures to the dorsum of the footpad at 31-cm source of surface distance). Immediately after irradiation, mice from related groups were injected subcutaneously with TLR9 agonist on the interscapular region with doses of 10 mg/kg body weight for monitoring of cytokines and immune response. For tumor studies, TLR9 agonist (1 mg/kg body weight) was injected subcutaneously, twice a week for three weeks. The tumor growth curve was generated by measuring three orthogonal tumor diameters (L×W×H) at 1–3-day intervals with a vernier caliper. Of the 75 wildtype mice, 40 mice were sacrificed 35 days after tumor inoculation and the remaining 35 mice were used for survival studies. Since 20 Gy of RT was not curative for these tumors, we performed below-knee amputation of the limb with tumors, 49 days after tumor innoculation for survival studies (see Figure 1B for detailed schema).

### Statistical Analysis

Statistical analyses were performed using Prism 4.0 software (GraphPad Software, San Diego, CA, USA). The difference of serum cytokine levels, lymphocyte frequencies and tumor growth rate among control and treated mice was analyzed by one-way ANOVA. Survival rate of mice after treatment was analyzed using Log-rank (Mantel-Cox) Test. A p value of <0.05 is considered significant.

## Results

### Immunomodulation by TLR9 Agonist in Tumor-bearing C57BL/6 Mice

TLR9 is expressed in B cells, pDCs and NKDCs. We examined whether the TLR9 ligand, TLR9 agonist could activate TLR9-expressing cells in vivo and analyzed the number and phenotype of splenic CD4+ T helper cells, CD8+ T cells, B cells, pDCs and NKDCs in tumor-bearing mice, 24 hours after TLR9 agonist administration (10 mg/kg body weight, s.c). Both active (TLR9 agonist) and nonactive (control oligo) compounds were tested in this study. As shown in Table 1, TLR9 agonist causes dramatic splenomegaly in mice (p<0.001) as compared with untreated and control oligo-treated mice, indicating an expansion of splenocytes with TLR9 agonist. Compared to untreated and control oligo-treated, tumor-bearing mice, TLR9 agonist selectively induced the proliferation of TLR9-expressing splenocytes, including B220+ B cells (p<0.001), Cd11c+B220+NK1.1-CD80+ activated pDCs (p<0.001) and CD11c+B220-NK1.1+ NKDCs (p<0.05) but reduced the percentage of CD3+CD4+ and CD3+CD8+ T cells (p<<0.001) (**Table 1**). These results are similar to the effect of TLR9 agonist in naïve mice without any tumors (data not shown) and confirm that TLR9 agonists activate TLR9+ cells with amplification of B cells, pDCs and NKDCs.

**Table 1:** TLR9 agonist induces the proliferation of TLR9-expression splenocytes in mice bearing an ectopic 3LL footpad tumor.

Treatment	Spleen Weight (g)	Percentage in total splenoctyes (%)				
		B cells	pDCs	NKDCs	CD3+CD4+	CD3+CD8+
NT	0.12±0.01	53.6±3.2	1.1±0.1	2.2±0.4	22.2±3.8	14.9±2.1
Control oligo	0.10±0.04	55.5±3.6	1.6±0.5	2.7±1.1	22.2±2.8	15.7±2.8
TLR9 agonist	0.46±0.04(***)^1^	71.7±8.8(***)	2.7±0.9(***)	3.7±0.8(*)	10.0±2.9(***)	6.9±1.5(***)
RT	0.30±0.04(***)	54.6±4.2	1.0±0.2	2.5±0.7	19.8±3.9	14.3±1.9
RT+TLR9 agonist	0.46±0.04(***)	72.9±3.0(**)	3.0±0.5(**)	5.0±0.7(**)	10.1±2.5(**)	6.5±1.7(***)

1 Significant different when compared with NT (No Treatment) group; p values are expressed as “***” (p<0.001); “**” (p<0.01) and “*” (p<0.05).

### Immunomodulation by RT+TLR9 Agonist in Tumor-bearing C57BL/6 Mice

Radiation is known to induce apoptosis in lymphocytes and other immune effector cells. Thus it is possible that RT could be deleterious for immune activation by TLR9 agonist. We therefore examined the effect of RT on various phenotypes of splenocytes. Previous report by Mason et al demonstrated that combination treatment with CpG oligonucleotides and a single fraction RT of 20 Gy was superior in tumor control, compared to a combination therapy of CpG and fractionated RT of 20 Gy [30]. In another report, single-dose RT was shown to be superior to fractionated RT in inducing tumor-specific T cells [9]. We, therefore, examined the immunomodulatory properties of a single fraction of 20 Gy delivered to a mouse Lewis lung adenocarcinoma, 3LL, grown on the dorsal aspect of the foot in C57BL/6 mice in combination with subcutaneous injection of TLR9 agonist.

**Figure 2:**
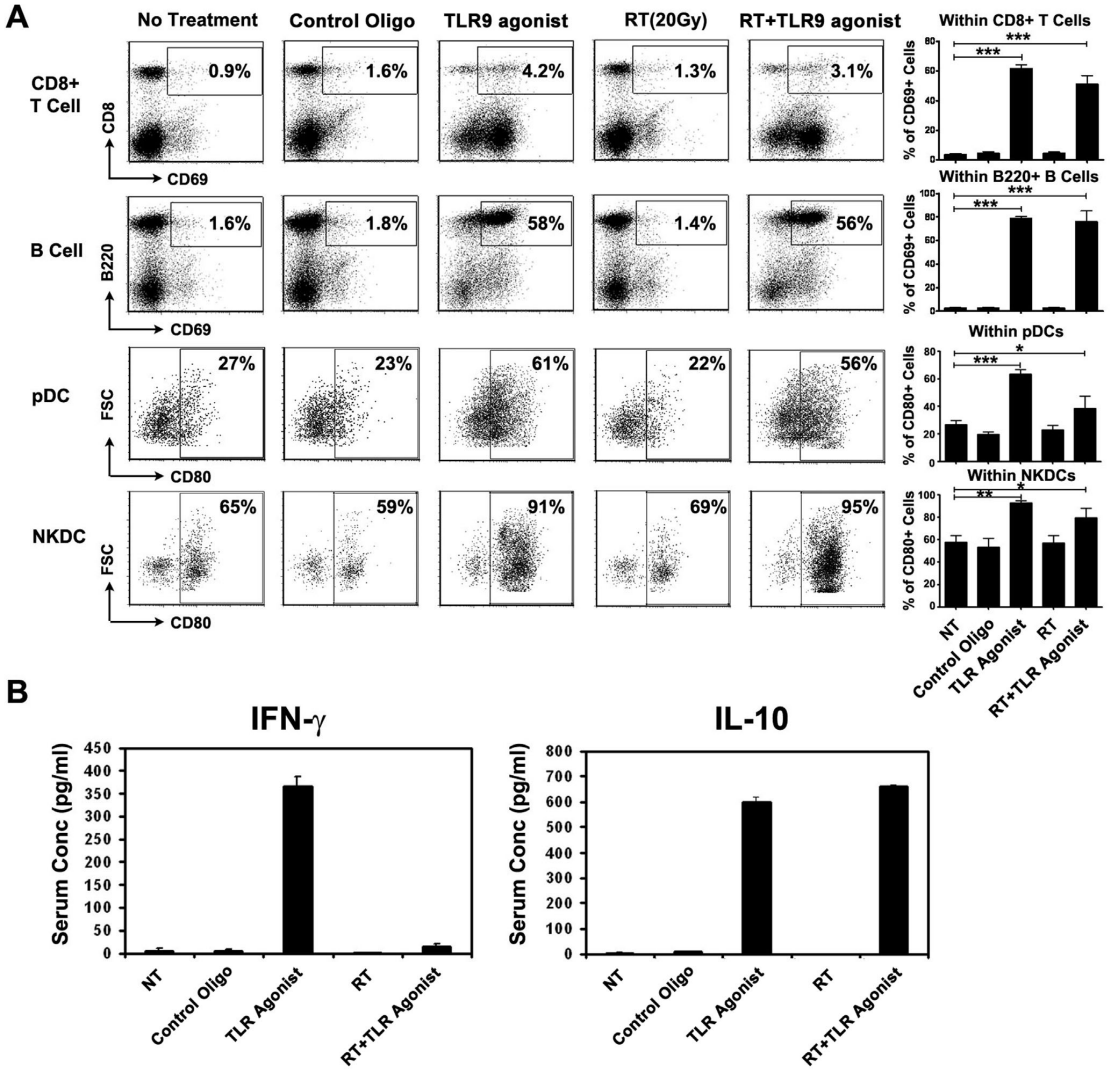
TLR9 agonist activates pDCs, NKDCs and B cells and increases the release of Th1 and Th2 cytokines in tumor-bearing C57BL/6 mice. C57BL/6 mice bearing 3LL tumor were divided into 5 groups receiving either PBS, control oligo, TLR9 agonist, RT (20 Gy) or RT+TLR9 agonist. Splenocytes and sera were harvested 24 hours after treatment. The activation of CD8+ T cells, B cells, pDCs and NKDCs was analyzed by flow cytometry (A) and the release of IFN-γ and IL-10 was analyzed by ELISA (B).

As shown in Table 1, animals treated with RT alone had larger spleen weight, compared to untreated controls (0.30±0.04 g in RT versus 0.12±0.01 g in untreated controls; P<0.001). However RT did not alter the percentage of T cells, B cells, pDCs and NKDCs, nor did RT induce their activation. Treatment with TLR9 agonist altered the relative percentage of splenocytes with potent proliferation of B cells, pDCs and NKDCs in animals that received combination RT and TLR9 agonist. There was no apparent difference of the immune phenotype of splenocytes between RT+TLR9 agonist and TLR9 agonist cohorts. Although the percentage of T cells was decreased after RT+TLR9 agonist treatment, TLR9 agonist activated CD8+T cells by upregualting the expression of CD69 on the cell surface. As shown in Figure 2A, more than half of the CD8+ T cells in the splenocyte population were dramatically activated and expressed the activation marker CD69+ after TLR9 agonist (61.7±5.7%) or RT+TLR9 agonist (51.4±7.8% CD69+) treatment. In contrast, only less than 5% of CD8+ T cells were activated in untreated and RT alone splenocytes. Besides activating CD8 T cells, TLR9 agonist treatment also activated B cells, pDCs and NKDCs, as evidenced by expression of activation markers, CD69 in lymphocytes and CD80 in DCs.

We next analyzed the release of Th1 and Th2 cytokines in the blood, 4 hours after RT and/or TLR9 agonist injection. As shown in Figure 2B, TLR9 agonist increased the serum levels of both IFN-γ (367±19 pg/ml) and IL-10 (598±130 pg/ml) as compared with untreated, control oligo and RT alone (all levels <10 pg/ml). Addition of RT induced a similar level of IL-10 release (662±44 pg/ml) but downregulated the secretion of IFN-γ (14.6±4.8 pg/ml). The decreased IFN-γ/IL-10 ratio indicates addition of RT to TLR9 agonist treatment induces polarization towards a Th2-mediated humoral immune response. This correlated with the proliferation and activation of B cells and reduced number of T cells in mouse spleen after RT+TLR9 agonist treatment.

### Local RT Augments the TLR9 Agonist-induced Tumor-reactive Humoral but not Cellular Immune response

**Figure 3:**
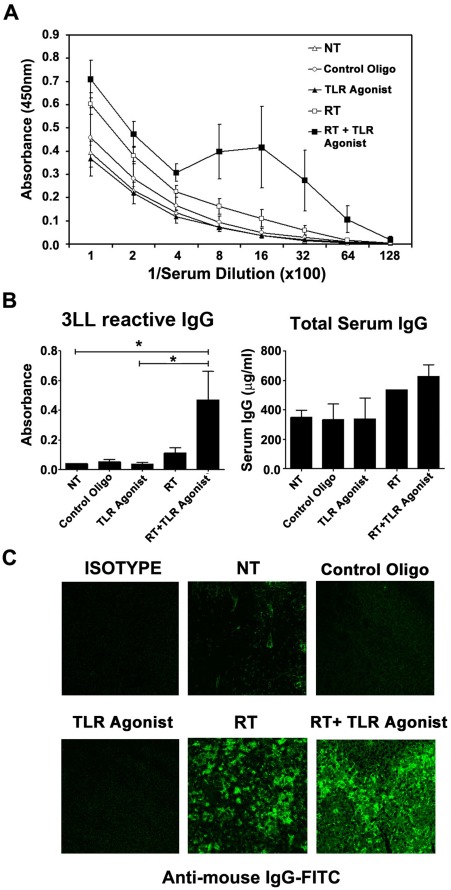
RT enhances TLR9 agonist induced tumor specific humoral immune response. Sera from tumor bearing mice treated with either PBS, control oligo, TLR9 agonist, RT (20 Gy) or combined RT and TLR9 agonist were analyzed for the presence of tumor specific antibodies by indirect ELISA coated with total 3LL tumor lysate (A). The total IgG titer was evaluated by an Easy-Titer Mouse IgG Assay Kit (B) and the infiltration of IgG antibody was visualized by confocal microscope after stained with FITC conjugated anti-mouse IgG (C).

We next determined whether combination treatment of RT+TLR9 agonist induces a systemic tumor-specific immune response in vivo. Mice with palpable 3LL tumors received 20 Gy single fraction RT and TLR9 agonist and control oligo twice a week for 3 weeks with subcutaneous injection. One week after last injection, mice were sacrificed and splenocytes and serum were collected for the detection of cellular and humoral immune response. As shown in Figure 3A, RT+TLR9 agonist treatment induced a potent IgG antibody response against 3LL tumor, as analyzed by indirect ELISA with 3LL tumor lysate coated as antigen on the ELISA plates. A weaker antibody titer was also observed in mice treated with RT alone while no significant antibody production was detected in untreated, TLR9 agonist and control oligo cohort. At a serum dilution of 1∶1600, RT+TLR9 agonist induced a significant level of tumor-reactive antibody, as compared with untreated (n = 8, p<0.05) and TLR9 agonist alone cohorts (n = 8, p<0.05). The total IgG titer was not significantly different among all the treatment groups with only slight increase in RT and combined RT+TLR9 agonist cohort (Figure 3B).

Since anti-tumoral IgG titers were induced after RT+TLR9 agonist treatment, we next examined whether these auto-antibodies were bound to the irradiated tumor tissue in situ. Fresh frozen sections of tumors were prepared from animals, 1 week after the last injection of TLR9 agonist and stained with hamster anti-mouse IgG. As negative control, we stained the fresh frozen tumor sections with FITC-labeled hamster IgG antibody. As seen in Figure 3C, RT increased the deposition of mouse IgG auto-antibodies on the tumor tissue, as noted by increased immunofluroscence in RT and RT+TLR9 agonist tumor cohorts. Tumor specimens from untreated and TLR9 agonist-treated animals exhibited very weak immunofluroscence, indicating no deposition of auto-antibodies in tumor tissue in situ. There was no binding of hamster IgG isotype control to the tumor specimen indicating that the direct immunofluroscence assay was specific. These results suggest that RT either induces tumor neoantigens or increase the distribution and binding of auto-antibodies to the tumor tissue.

We further analyzed the tumor-specific cellular immune response in these animals. Splenocytes were cocultured with irradiated 3LL tumor cells or incubated with a 3LL-specific tumor antigenic peptide MUT1 or PMA+Ionomycin for 40 hours and the release of IFN-γ was detected by ELISpot assay. No tumor specific IFN-γ release was observed in any cohort, suggesting the absence of a measurable cellular T cell response (see Figure S1). These results correlate with decreased IFN-γ/IL-10 ratio observed in mice treated with RT+TLR9 agonist and confirm the potential immunomodulatory role of single fraction RT in polarizing a Th2-mediated humoral immune response.

### RT+TLR9 Agonist Treatment Inhibits Tumor Progression in both C57BL/6 and Congenic B Cell Deficient Igh-6^tm1Cgn^ Mice

**Figure 4:**
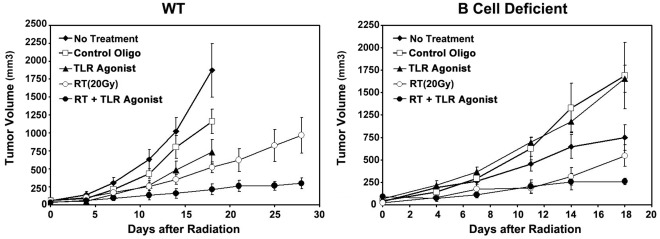
RT+TLR9 agonist treatment inhibits tumor progression in both C57BL/6 and congenic B cell deficient mice. Wild type (C57BL/6) or congenic B cell deficient mice (Igh-6^tm1Cgn^) bearing 3LL tumors were divided into 5 groups receiving either PBS, control oligo, TLR9 agonist, RT (20 Gy) or RT+TLR9 agonist. Tumor growth curves were generated by measuring three orthogonal tumor diameters at 1–3-day intervals with a vernier caliper.

To evaluate the therapeutic effect of RT+TLR9 agonist treatment on the tumor, a tumor growth curve was generated by measuring the tumor volume at 1–3-day intervals. As shown in Figure 4, significant tumor growth retardation was seen in mice treated with RT+TLR9 agonist as compared with untreated mice (p<0.01). Animals treated with RT or TLR9 agonist alone also exhibited initial tumor growth retardation in the first two weeks post-treatment but the tumors eventually regrew after treatments were stopped. To further validate whether this anti-tumor effect was mediated by the tumor-specific humoral response generated by RT+TLR9 agonist, the tumor growth study was performed in congenic B cell deficient mice, which lacks the ability to produce antibody. Surprisingly, RT+TLR9 agonist still induced significant tumor growth retardation as compared with untreated mice (p<0.01). However, there was no difference between RT alone and RT+TLR9 agonist cohort during the first 11 days indicating a dominant RT effect over TLR9 agonist. The tumor treated with RT alone regrew after 14 days while tumor treated with RT+TLR9 agonist remained stabilized. This result suggests that mediators other than antibodies might be also involved in the generation of anti-tumor effect by RT+TLR9 agonist.

### RT Enhances the Infiltration of TLR9 Agonist-activated NKDCs in Tumor Tissues

**Figure 5:**
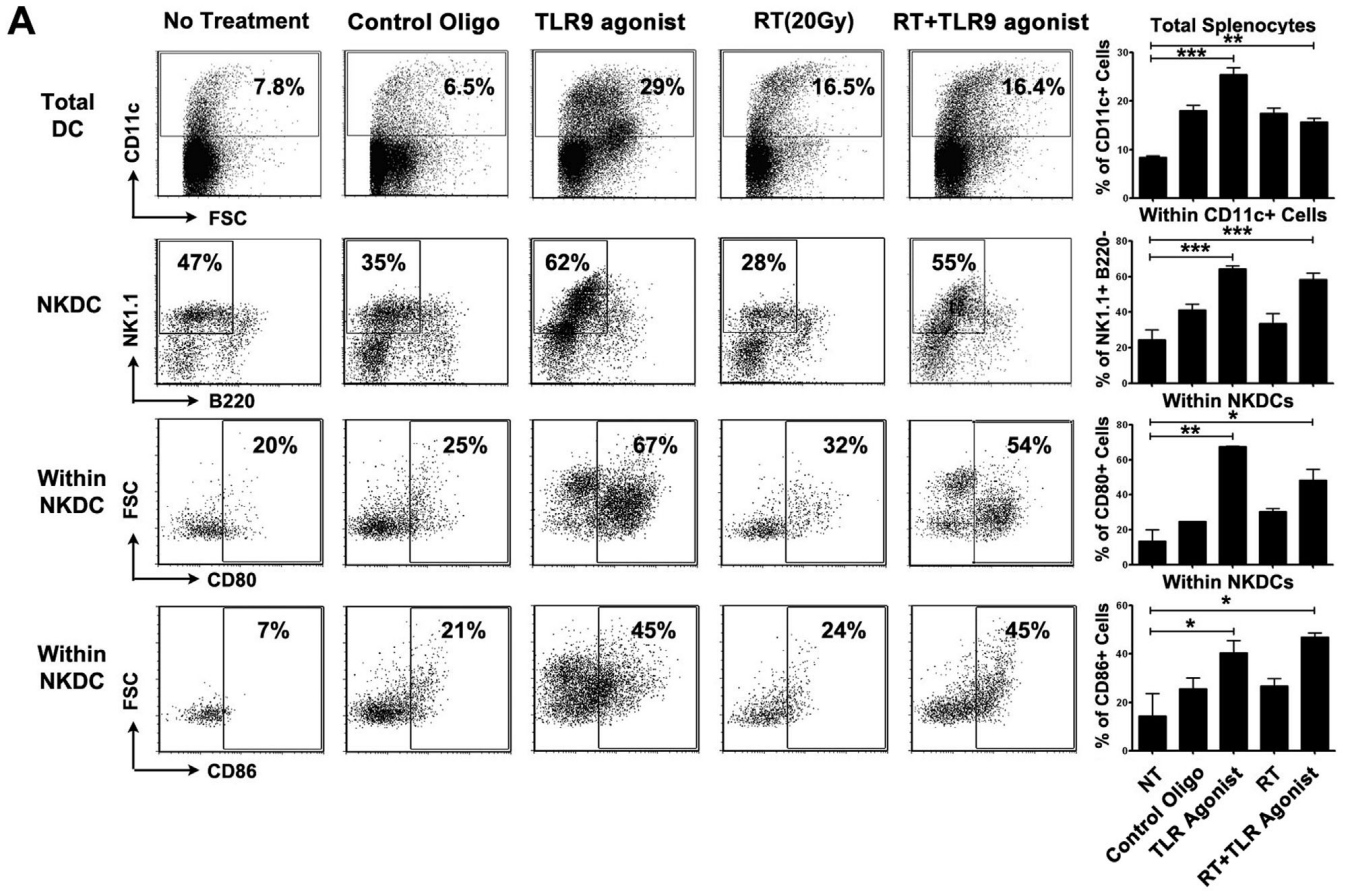
RT induced the proliferation and activation of NKDCs in congenic B cell deficient mice. Splenocytes from B cell deficient mice treated with either PBS, control oligo, TLR9 agonist, RT (20 Gy) or RT+TLR9 agonist were stained with anti-CD11c, NK1.1, B220, CD80 and CD86 antibodies and the percentage of activated NKDCs in total DCs were analyzed by flow cytometry. The difference among each treated group was analyzed by one-way ANOVA.

**Figure 6:**
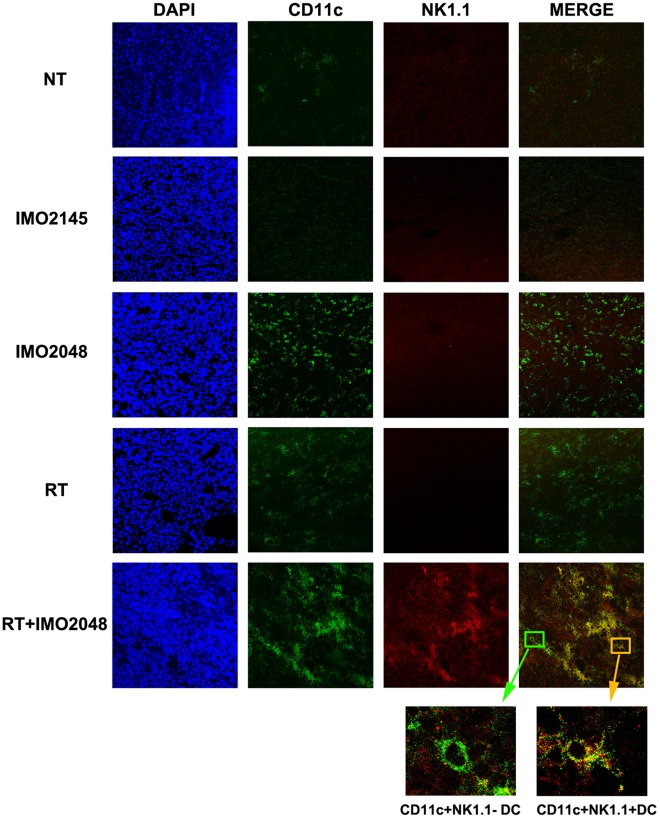
RT induced the penetration of NKDCs in tumor tissue. Tumor samples from B cell deficient mice treated with either PBS, control oligo, TLR9 agonist, RT (20 Gy) or RT+TLR9 agonist were stained with anti-CD11c and NK1.1 antibodies and the presence of NKDCs was visualized by confocal microscope.

In B cell deficient mice, B cells and pDCs are only less than 1.5% of the whole splenocytes population, while T cells and NK cells consist of over 60% of the splenocytes (See Table S1). To investigate the mechanism of TLR9 agonist+RT induced anti-tumor effect in these mice, we first examined whether tumor-specific T cell immune response is induced by IFN-γ ELISPOT assay. As noted in the wild-type mice, these mice did not develop a detectable T cell-mediated immune response to 3LL cells in any of the treatment cohorts (see Figure S1). We, therefore, examined the immunoprotective role of CD11c+B220-NK1.1+ NKDCs, which also express TLR9 in B cell-deficient mice. While NKDCs consist of 25.4±11.1% of the splenic DC population in these mice, TLR9 agonist treatment significantly increased the proportion of CD11c+B220-NK1.1+ NKDCs (Figure 5. TLR9 agonist, 64.4±3.4% vs. untreated, 24.33±9.7%, p<0.001) while reducing the percentage of conventional CD11c+B220-NK1.1- DCs (30.9±9.4%) (See Table S2). Although RT alone resulted in a slight increase in NKDCs (RT, 33.6±8.1% vs. untreated; p>0.05, ns), addition of TLR9 agonist to RT increased the percentage of NKDCs to significantly higher levels (RT+TLR9 agonist, 58.6±5.8% versus untreated; p<0.001). The activation phenotype of NKDCs from mice treated with RT or control oligo were not different from those from the untreated mice. However, 45–67% of the NKDCs from TLR9 agonist-treated mice upregulated the expression of the activation markers for DCs, CD80 and CD86 (Figure 5), indicating that TLR9 agonist treatment resulted in proliferation and activation of NKDCs. Next we analyzed whether RT could facilitate the infiltration of TLR9 agonist-activated NKDCs in the irradiated tumor tissues. As shown in Figure 6, RT+TLR9 agonist treatment induced a significant infiltration of NKDC in the tumor tissue while TLR9 agonist and RT alone induced a slight infiltration of only conventional DCs. The selective proliferation, activation and infiltration of NKDCs after TLR9 agonist treatment suggests that these immune effector cells might have direct contribution to the tumoricidal effects of combination treatment in B cell-deficient mice.

**Figure 7:**
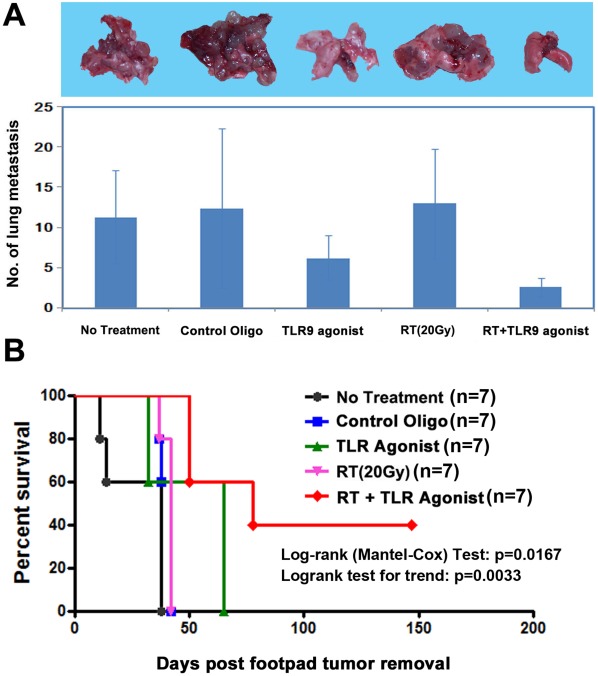
RT+TLR9 agonist treatment prevents tumor metastasis and improves survival. The number of lung metastase in wild type C57BL/6 mice treated with either PBS, control oligo, TLR9 agonist, RT (20 Gy) or RT+TLR9 agonist was counted and compared (A). The survival rate of mice from each group after treatment was analyzed using Log-rank (Mantel-Cox) Test (B).

### RT+TLR9 Agonist Treatment Prevents Tumor Recurrence and Metastasis and Improves Survival

Since mouse with Lewis lung adenocarcinoma develop spontaneous pulmonary metastases, we investigated whether combination treatment could prevent tumor recurrence and metastases. In order to study the effect of systemic metastases on survival, the primary tumors were removed from all animal cohorts (n = 7/group), 49 days after tumor inoculation (see Figure 1B). As shown in Figure 7A, animals from untreated, control oligo and RT cohorts developed gross pulmonary metastases and died within 50 days after tumor removal. In contrast, RT+TLR9 agonist treatment significantly reduced the number of lung metastases and improved survival (median survival time RT+TLR9 agonist, 78 days vs. untreated 38 days; p<0.005 log-rank test; Figure 7B) with 40% of the animals surviving more than 150 days. These results indicate that combined TLR9 agonist and RT treatment could provide both local and systemic protection against 3LL tumor.

## Discussion

TLR9 agonists have been widely used in cancer therapy due to its ability of inducing potent anti-tumor immune response [12]. In the present study, we have evaluated the potential of combining single fraction RT with a new generation of synthetic agonist of TLR9, consisting of a 3′-3′-attached structure and a CpR motif that shows potent immunostimulatory effects in diverse vertebrate species, including human, monkey, pig, horse, sheep, goat, rat, and chicken [34]. This type of oligonucleotides has great advantage over conventional CpG oligonucleotides because of the poor responsiveness of CpG ODN in human clinical trials. By combining TLR9 agonist with RT, we demonstrate a potent synergistic local and systemic tumoricidal effect in mice with 3LL tumors. Consistent with previous studies, TLR9 agonist induced spleen enlargement in mice and induced the proliferation of TLR9-expressing splenocytes, including, B220+ B cells, Cd11c+B220+NK1.1-CD80+ pDCs and CD11c+B220-NK1.1+ NKDCs. Furthermore, it induced the activation of B and T cells and pDCs with release of IFN-γ and IL-12 in the serum. Interestingly, addition of primary tumor RT caused minimal changes in the phenotype of splenocytes suggesting that RT does not suppress the immunomodulatory effects of TLR9 agonist. However, the serum cytokine profile was different between RT and RT+TLR9 agonist cohorts. RT reduced the levels of serum IFN-γ while maintaining the serum IL-10 levels, compared to animals treated with RT+TLR9 agonist. These results suggest that while TLR9 agonist has been shown to induce both Th1 and Th2 immune response, single fraction 20 Gy to the tumor might shape the immune response toward a Th2-bias, which facilitates the generation of tumor-reactive antibodies. Consistent with this finding, a potent tumor-reactive antibody response was found in mice receiving RT+TLR9 agonist treatment, while no detectable tumor-reactive antibody titer was found in other cohorts. However, the total serum IgG titer was not found significantly different among various treatment cohorts. Thus single fraction RT appears to generate a tumor-reactive humoral immune response without influencing the total amount of antibody generated. Furthermore, fluorescenct immunohistochemistry demonstrated deposition of mouse IgG auto-antibodies in tumor tissue in situ following RT.

Surprisingly, the tumoricidal effects of RT+TLR9 agonist combination therapy were maintained in B cell-deficient mice, suggesting that immune cells, other than B cells, contribute to the control of tumor progression. Since TLR9 is also expressed on NKDCs, we analyzed the phenotype of these cell subsets in mice receiving combination therapy. TLR9 agonist significantly increased the proportion of NKDCs in DC population while decreased the percentage of conventional DCs. Addition of RT slightly downregulated the effect of TLR9 agonist while still demonstrating a significant upregulation, compared to untreated mice. Furthermore, TLR9 agonist activated NKDCs by upregualting the expression of both CD80 and CD86, while RT increased the tumor infiltration of NKDCs. Since NKDCs were reported as a potent effect cell population that has direct cytotoxic effect against tumor cells [16], [35], these results indicate NKDCs might contribute to the anti-tumor effects, induced by combined TLR9 agonist and RT treatment. The mechanism of RT-induced recruitment of lymphocytes and antibodies to tumor tissue is still unclear. Previous studies showed that RT upregulated the expression of VCAM-1 on the tumor vasculature [7] and induced the release of chemokines, such as CXCL16 from tumor cells [8], which may favor the trafficking of immune effector cells to the tumor tissue.

The effect of RT dose and fractionation in immunomodulation is unclear. Previous studies have shown that large single fraction of RT (15–20 Gy) is effective in immunomodulation with CpG oligonucleotode immunotherapy for murine fibrosarcoma [30]. Similarly, a single fraction of 15 Gy induced melanoma-specific T cells and promoted intratumoral infiltration [9]. In contrast, fractionated RT was found to be more effective than single fraction RT in tumor control when combined with anti-CTLA4 antibody for a murine model of breast tumor [36]. However, the last report used hypofractionated doses (6–8 Gy) during the fractionated RT and thus it is possible that an ablative, large single fraction (15–20 Gy) of RT or hypofractionated RT can induce anti-tumoral immune response more effectively than conventional fractionated RT (1.8–2 Gy single fraction). Possible explanation might be that large fraction size induces translocation of cytosolic calreticulin to the cell surface that promotes phagocytosis of irradiated tumor cells by tumor infiltrating dendritic cells [37]. Therefore, ablative RT might induce an immunogenic tumor cell death that can release tumor antigens, which are actively phagocytosed by DCs and provide danger signals for DC activation and induction of immune response [37]. It is possible that primary tumor RT effectively generates a local tumor antigen pool for APCs [38], such as, TLR9 agonist-activated pDCs, which then uptake the tumor antigens and migrates to the lymph nodes where it stimulates the Th2 cells to elicit a humoral immune response. In addition, the danger signals induced by RT in dying tumor cells and the modulation of intratumoral cytokine melieu potentially plays a critical role in eliciting tumor-specific immune response. Further studies are ongoing on how RT modifies the tumor microenvironment to favor the induction of tumor specific immune response.

In summary, the immunomodulatory effects of RT significantly enhance the efficacy of immunotherapy with TLR9 agonists. RT+TLR9 agonist therapy induced tumor-specific IgG auto-antibodies, and stimulated proliferation and activation of TLR9-expressing B cells, pDCs and NKDCs. The immunomodulatory effects of the combination therapy improved local control of RT and reduced systemic pulomonary metastases, thus providing a strong rationale for combining RT with TLR9 agonist immunotherapy for improved loco-regional and systemic tumor therapy. Finally, we recently demonstrated that systemic administration of the synthetic TLR9 agonist protects against intestinal injury and mucositis in radiation-induced gastrointestinal syndrome, without conferring any radioprotection to abdominal tumors [39]. Thus, TLR9 agonist could improve the therapeutic ratio of RT by providing radioprotection of normal moucosal tissues, while increasing the tumoricidal effects of primary tumor RT and induction of systemic anti-tumoral immunity for eradication of systemic metastases.

## Supporting Information

Figure S1 **Detection of T cell response in mice treated with TLR9 agonist and/or RT by ELISpot assay.** Splenocytes from control wildtype and B cell deficient mice or mice treated with either TLR9 agonist, control oligo, RT or combined RT and TLR9 agonist were cocultured with either medium, PMA+Ionomycin or 3LL cells. The 3LL-specific IFN-γ releasing splenocytes were detected by ELISpot assay.
(TIF)

Table S1 **Lymphocyte population in wildtype and B cell deficient mice (n = 5).** Splenocytes from naïve wildtype and B cell deficient mice were stained with fluorophore conjugated antibodies against CD3, B220, NK1.1 and CD11c and were analyzed by flow cytometry. Percentage of each lymphocyte subset was calculated using Flowjo software.
(DOCX)

Table S2 **Percentage of DC subsets in B cell deficient mice treated with TLR agonist and/or RT.** Splenocytes from control B cell deficient mice or mice treated with either TLR9 agonist, control oligo, RT or combined RT and TLR9 agonist were stained with fluorophore conjugated antibodies against B220, NK1.1 and CD11c. Stained cells were analyzed by flow cytometry and percentage of each DC subset was calculated using Flowjo software.
(DOCX)
