# Supplementary figures and images for: An In Situ Autologous Tumor Vaccination with Combined Radiation Therapy and TLR9 Agonist Therapy
Source: PLoS One. 2012 May 30;7(5):e38111. doi: 10.1371/journal.pone.0038111 (PMC3364192; doi:10.1371/journal.pone.0038111)

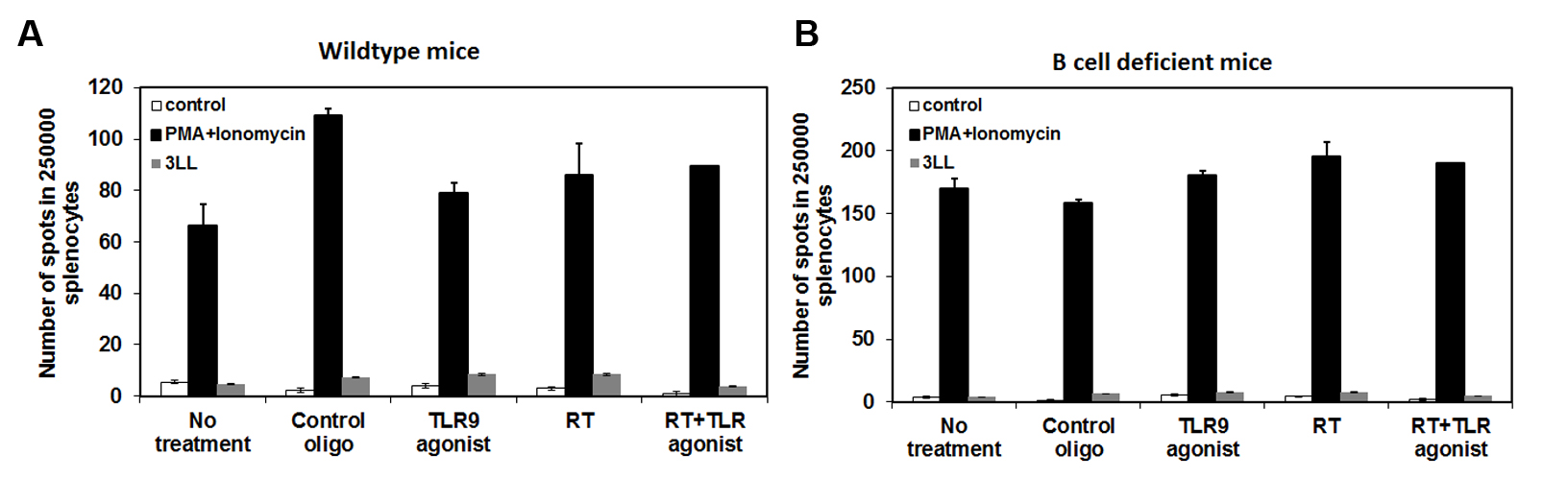

Supplement: Figure S1 — Detection of T cell response in mice treated with TLR9 agonist and/or RT by ELISpot assay. Splenocytes from control wildtype and B cell deficient mice or mice treated with either TLR9 agonist, control oligo, RT or combined RT and TLR9 agonist were cocultured with either medium, PMA+Ionomycin or 3LL cells. The 3LL-specific IFN-γ releasing splenocytes were detected by ELISpot assay. (TIF) [file pone.0038111.s001.tif]
