# Supplementary material for: An In Situ Autologous Tumor Vaccination with Combined Radiation Therapy and TLR9 Agonist Therapy
Source: PLoS One. 2012 May 30;7(5):e38111. doi: 10.1371/journal.pone.0038111 (PMC3364192; doi:10.1371/journal.pone.0038111)
Supplement: Table S1 — Lymphocyte population in wildtype and B cell deficient mice (n = 5). Splenocytes from naïve wildtype and B cell deficient mice were stained with fluorophore conjugated antibodies against CD3, B220, NK1.1 and CD11c and were analyzed by flow cytometry. Percentage of each lymphocyte subset was calculated using Flowjo software. (DOCX) [file pone.0038111.s002.docx]

Table S1. Lymphocyte population in wildtype and B cell deficient mice (n=5).

| Cell population | Wild type | B cell deficient |
| --- | --- | --- |
| T cells (CD3+) | 37.2±5.2* | 54.1±26.2 |
| B cells (B220+) | 53.6±3.2 | 1.2±0.7 |
| NK cells (CD1.1+) | 7.6±0.8 | 16±10.6 |
| DCs (CD11c+) | 5.8±1.0 | 7.1±2.7 |
| pDCs(CD11c+B220+NK1.1-) | 0.8±0.1 | 0.3±0.1 |
| NKDCs (CD11c+B220-NK1.1+) | 1.9±0.4 | 2.0±1.4 |

* Number represents the percentage of a subset in total splenocytes.
