# Supplementary material for: An In Situ Autologous Tumor Vaccination with Combined Radiation Therapy and TLR9 Agonist Therapy
Source: PLoS One. 2012 May 30;7(5):e38111. doi: 10.1371/journal.pone.0038111 (PMC3364192; doi:10.1371/journal.pone.0038111)
Supplement: Table S2 — Percentage of DC subsets in B cell deficient mice treated with TLR agonist and/or RT. Splenocytes from control B cell deficient mice or mice treated with either TLR9 agonist, control oligo, RT or combined RT and TLR9 agonist were stained with fluorophore conjugated antibodies against B220, NK1.1 and CD11c. Stained cells were analyzed by flow cytometry and percentage of each DC subset was calculated using Flowjo software. (DOCX) [file pone.0038111.s003.docx]

Table S2. Percentage of DC subsets in B-/- mice treated with TLR agonist and/or RT.

| Treatment  (n=5/group) | Conventional DCs (CD11C+B220-NK1.1-) | Plasmacytoid DCs (CD11c+B220+NK1.1-) | NKDCs  (CD11c+B220-NK1.1+) |
| --- | --- | --- | --- |
| NT | 65.6±8.3* | 5.1±1.3 | 24.3±9.7 |
| Control oligo | 48.2±6.6 | 4.8±2.5 | 41.3±5.7 |
| TLR9 agonist | 30.9±9.4 (***) | 1.9±0.5 | 64.4±3.4(***) |
| RT | 39.1±3.0 (***) | 2.4±1.0 | 33.6±8.1 |
| RT+TLR9 agonist | 33.9±6.9 (**) | 1.7±0.7 | 58.6±5.8(***) |

*Number represents the percentage of a subset within total splenic DCs (CD11c+).
